# Theoretical study on the role of dynamics on the unusual magnetic properties in MnBi

**DOI:** 10.1038/srep07222

**Published:** 2014-11-27

**Authors:** K. V. Shanavas, David Parker, David J. Singh

**Affiliations:** 1 Oak Ridge National Laboratory, 1 Bethel Valley Road, Oak Ridge, Tennessee 37831, USA

## Abstract

We study the electronic structure and lattice dynamics in the ferromagnet MnBi using first-principles calculations and a tight-binding model. The band structure around the Fermi level is dominated by Bi-*p* states which are the primary contributors to the magnetic anisotropy energy in the low temperature structure. A tight-binding model consisting of Mn-*d* and Bi-*p* states is developed and the parameters are determined from first-principles calculations. Phonon dispersions and elastic moduli exhibit several interesting features. The results imply that the magnetic interaction with the crystal lattice in MnBi is considerably more complex than previously thought and in particular that there is a rich interplay between phonons and magnetism involving both magnetoelastic and magnetostrictive coupling.

In recent years, significant attention has been paid to realize permanent magnets without rare earth elements. Due to its large uniaxial magnetic anisotropy energy (MAE) and Curie temperature above room temperature, MnBi has emerged as a favorable candidate among transition metal systems^1^,^2^,^3^. In addition, it has good magneto-optical properties including a large Kerr rotation^4^ with potential applications in erasable magneto-optical memory devices^5^.

In the low-temperature phase, MnBi is a ferromagnetic compound with the NiAs structure. Above 628 K, a first-order paramagnetic transition accompanied by a phase decomposition to Mn_1.08_Bi takes place^6^. The magnetic phase of MnBi is unusual in that the anisotropy constant (*K_u_*) increases with temperature unlike most magnets^2^. Also, *K_u_* is negative below ~90 K, with all the magnetic moments aligned in the in-plane direction. A spin-reorientation transition takes place at *T*_SR_ ≈ 90 K, when the magnetization changes direction towards *c* axis. Thus, *K_u_* starts at −0.2 MJ/m^3^ close to 0 K and reaches 2 MJ/m^3^ at room temperature. The lattice parameters also exhibit a small kink at the spin-reorientation temperature indicative of a first order transition^7^. Mechanical grinding of the crystalline samples were found to enhance room temperature coercivity, at the same time suppressing the spin-reorientation temperature^8^ which was attributed to the competition between second and higher order crystal field terms. MnBi also exhibits a large Kerr effect, with a double peak spectrum similar to other ferromagnetic transition metals^4^,^9^. Interband transitions between Mn 3*d* and Bi 6*p* states were suggested to be responsible for the effect.

Several theoretical studies have been carried out to understand the magnetic properties of MnBi^3^,^10^,^11^,^12^,^13^. Electronic structure calculations correctly reproduce the low temperature magnetic structure, which is ferromagnetic and metallic^14^. Calculated *K_u_* is negative, in agreement with experiments, but it's magnitude is overestimated. Sakuma *et al* found that a slight decrease in the valence electron number can change the sign of *K_u_*, so that partially substituting Bi with Sn (MnBi_1−*x*_Sn*_x_*) can lead to the positive anisotropy observed at high temperatures^11^. The volume derivative of *K_u_* is also negative in density functional theory (DFT) based calculations, but including Coulomb correlations through DFT + *U* makes it positive^12^,^13^. Thus, thermal expansion of the lattice was suggested as a potential reason for the spin-reorientation transition by Zarkevich *et al*, who found MAE to change sign for larger values of the lattice parameter *a*^12^. More recently, Antropov *et al*^13^ studied the spin-reorientation in MnBi with the help of extensive LSDA + *U* calculations, in which they treated *U* as an adjustable parameter and found good agreement with experimental observations for a larger value of *U*. Since, MnBi is not expected to be a strongly correlated system, it is surprising that anisotropy energies depend strongly on *U*.

In MnBi, substantial coupling of magnetism with the lattice is implied, which may lead to interesting lattice dynamical behavior in relation to magnetism. Thermal vibrations of atoms can alter the crystal field of the ions which can in turn affect the single ion anisotropy. In addition, spin-fluctuations lead to temperature dependence of the molecular field, also affecting the anisotropy. These effects have been suggested as responsible for spin-reorientation in rare earth magnets such as Nd_2_Fe_14_B^15^. Hence, in this paper, we explore the role of lattice dynamics on the magnetic behavior of MnBi with the help of first principles calculations. We also present a simple tight-binding model for the low temperature structure involving 3*d* and 6*p* electrons, which can reproduce the magnetic ground state. We find that calculated phonon spectra and sound speeds in MnBi are high anisotropic. Our calculations suggest that thermally induced lattice vibrations contribute significantly to the spin-reorientation transition in addition to thermal expansion.

## Results

### Effect of Hubbard *U*

**Figure 1:**
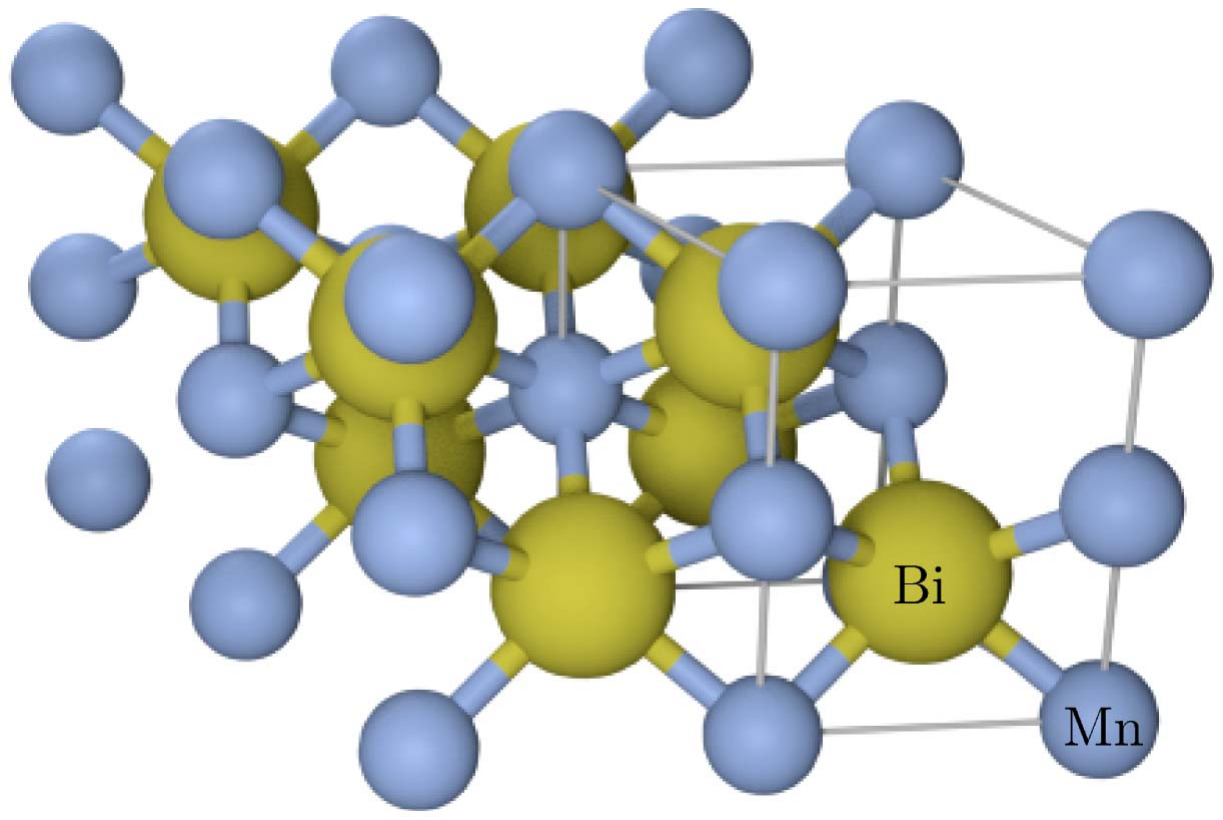
The hexagonal crystal structure of MnBi. The Mn and Bi ions are arranged in alternating layers along *c* axis each having six nearest neighbors. The unitcell contains two formula units.

In the low temperature phase, MnBi crystallizes in the hexagonal structure with space group 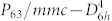, with the unit cell as shown in Fig. 1. The experimental structure from Ref. 6 is used in our calculations, with lattice constants *a* = *b* = 4.285 Å and *c* = 6.113 Å. Along the *c* direction, Mn and Bi are arranged in alternating layers, each bonded to six nearest neighbors. The Bi sites have a mirror plane, while Mn sites have inversion center as can be seen from Fig. 1. The valence atomic configuration of Bi is 6*s*^2^6*p*^3^ and that of Mn is 3*d*^5^4*s*^2^.

**Figure 2:**
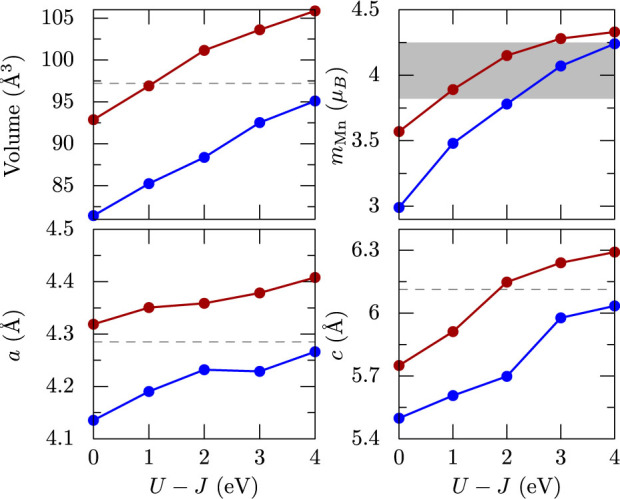
Effect of Coulomb repulsion *U* on the structure and magnetism of MnBi from LDA + U (blue lines) and GGA + U (red lines) calculations obtained through structural optimizations in the spin-polarized phase. The experimental lattice parameters from Ref. 6 are shown as horizontal dotted lines and the measured Mn magnetic moments from Ref. 17 (3.82 *μ_B_*) and Ref. 7 (4.25 *μ_B_*) are marked as the shaded area.

Since strong correlations are suggested to play an important role in the properties of MnBi, we systematically study the effect of different exchange correlations (LDA and GGA) and *U* parameter values (0–4 eV) on the structure and magnetism. A rotationally invariant implementation of *U* is used in the calculations^16^ and the results are summarized in Fig. 2.

We find that, in the *U* = 0 eV limit, both LDA and GGA underestimate the volume with 16.6% and 4.4% errors respectively. The better agreement between GGA and experiments is because it gets the *a* lattice parameter more or less correct, while LDA underestimates both. Magnetic moments of Mn atoms are better with GGA and including a small *U* of 1–2 eV in GGA + *U* calculations improves *c* and *m*_Mn_, but agreement in *a* worsens. On the other hand, in the case of LDA, Hubbard *U* of more than 4 eV is necessary to reach good agreement with experiments, as can be seen from Fig. 2.

For comparison, we also calculated the optimized lattice parameters and magnetic moments using more accurate all-electron code WIEN2k within LDA, GGA-PBE and GGA-PBEsol approximations. The results are summarized in Table I. The LDA and GGA results are consistent with those form VASP calculations in Fig. 2. Interestingly, PBEsol does not substantially improve the results in this system.

### Electronic structure

**Figure 3:**
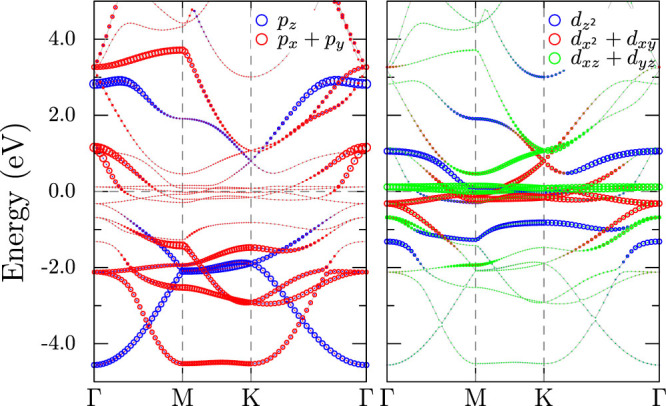
Electronic band structure of MnBi calculated using GGA in the paramagnetic phase. The orbital character of Bi-*p* states (left) and Mn-*d* states (right) are shown as colored circles, with the sizes proportional to the weight.

The non-spin polarized GGA band structure of MnBi with the experimental structure are shown in Fig. 3. The bands close to the Fermi level are made up of Bi-*p* and Mn-*d* orbitals. The Bi-*s* band lies deeper (not shown), around −10 eV and the Mn-*s* orbitals are empty. These results suggest that the valence configuration is close to 3*d*^6^6*p*^4^ where the Mn 4*s* electrons are shared between Mn and Bi. The Bi-*p* bands have large energy dispersion, spanning from −4.5 eV to 4 eV while the Mn-*d* bands are narrow (from −2 eV to 1.5 eV). Among the *d* orbitals, 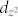 has the largest dispersion.

**Figure 4:**
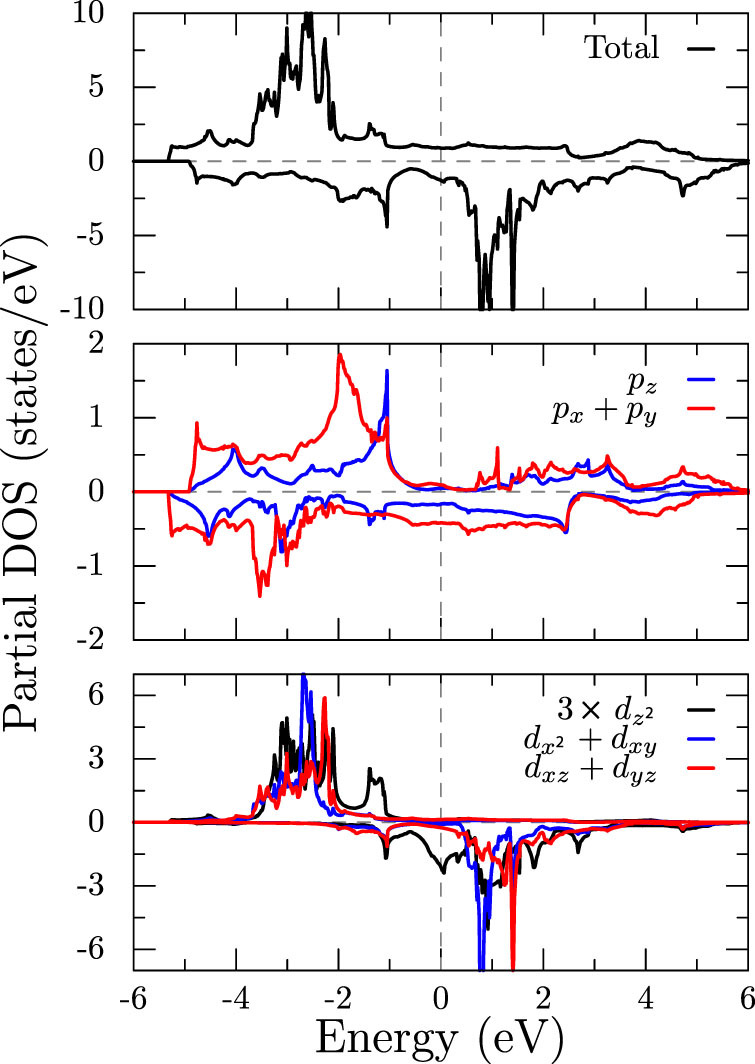
Total and partial density of states from the spin-polarized GGA calculation for MnBi. Positive and negative values of DOS correspond to spin-up and spin-down respectively. Fermi level is at 0 eV.

With spin polarization, the up and down *d* spin states are split by an exchange field of ~3.5 eV, as evident from the density of states in Fig. 4. The majority channel of Mn-*d* orbitals are fully occupied, however the minority channel is partially occupied by ~0.8 electrons per Mn, leading to *d*^5.8^ configurations at the Mn sites and a magnetic moment close to 4 *μ_B_*. An opposite spin-polarization of about −0.15 *μ_B_* is induced at the Bi sites as can be seen from the middle panel of Fig. 4. The calculated band structure and DOS compare reasonably well with previous calculations^10^,^14^. Also, experimental measurements using photoelectron spectroscopy^18^ suggest that the occupied electron density peaks around −3 eV below the Fermi level, which is in agreement with Fig. 4.

We find that, when spin-orbit coupling is included in the calculations, the energy of the system is lower by 1.2 meV/cell with the magnetic moments pointing along the *a* axis than when moments point along *c* axis. This leads to a MAE constant *K_u_* = −1.97 MJ/m^3^, which is larger than the experimental value of −0.2 MJ/m^3^, but consistent with earlier theoretical calculations within GGA. Repeating the calculation for the moments pointing along [101] direction and using the expansion of anisotropy for hexagonal crystals, viz., *K* = *K*_1_ sin^2^*θ* + *K*_2_ sin^4^*θ* + …, we can estimate the leading terms. We get, *K*_1_ = −1.2 meV and *K*_2_ = 0.009 meV, which show that *K*_2_ is too small to play any significant role in this system.

### Tight-binding model

**Figure 5:**
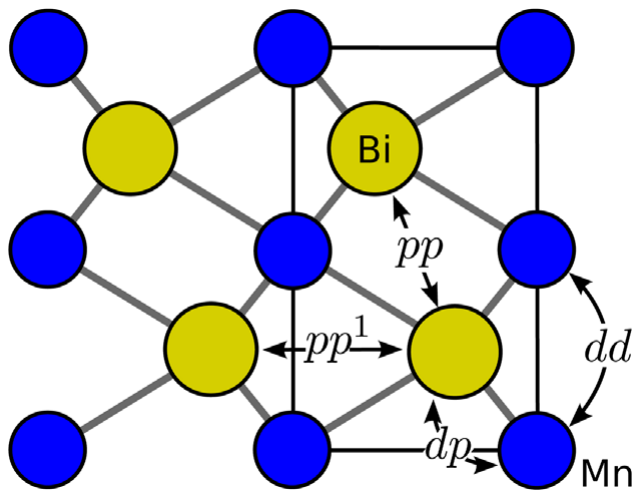
Electron hopping in MnBi considered for the tight-binding model. Being a bigger atom, Bi has coupling with nearest and next nearest neighbor Bi atoms in addition to Mn. There is weak *dd* coupling along *c* direction.

A linear combination of atomic orbitals are used as the basis for constructing the tight-binding (TB) Hamiltonian and the periodic nature of the crystal is taken into account through the Bloch sum. From first-principles calculations discussed in the previous section, we know that the bands near Fermi level are made up of predominantly Bi-*p* and Mn-*d* states. Since there are two formula units in the primitive cell, the Hamiltonian spans 16 × 16 in orbital space and 2 × 2 in spin space. It has the following three component form, 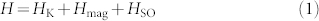
The first term, *H*_K_ contains the electron kinetic energy and has same form for both spins. It can be written in terms of onsite energies *ε_α_* and Slater-Koster hopping integrals *E_α_*_,*β*_(***ρ****_ij_*) within two-center approximation. Denoting the basis functions as |*iασ*〉, where *i*, *α* and *σ* correspond to site, orbital and spin indices, we can write 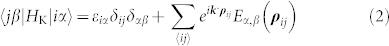
where, ***k*** is the wave vector and ***ρ****_ij_* is the vector connecting the neighbors *i* and *j*. For MnBi, we considered contributions from all neighbors and found that in addition to the nearest neighbor *d* − *p* coupling arising from Mn-Bi bonds, we also need to consider Mn-Mn interaction along *c* axis, nearest and next-nearest neighbor Bi-Bi interactions as shown in Fig. 5. Second neighbor coupling for Mn are weak and can be ignored to the lowest order.

After performing the sum over the neighbors indicated in Fig. 5, the second part of Eq. 2 can be written as, 
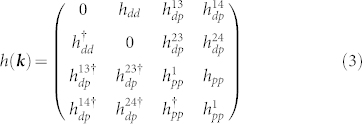
The hopping matrices *h_dd_*, *h_dp_* and *h_pp_* are given in the Appendix. They depend on the wave vector ***k*** and the seven Slater-Koster parameters: *t_ddσ_*, *t_ddπ_*, *t_ddδ_*, *t_dpσ_*, *t_dpπ_*, *t_ppσ_*, *t_ppπ_*. The onsite energies *ε_i_* are taken to be equal. Ignoring any spin-dependence of these parameters, we use *ab-initio* calculations with the paramagnetic structure to derive them. For hopping parameters, we assume a power-law distance dependence, *t*(*r*) = *t*(*r*_0_)(*r*_0_/*r*)*^n^*, where *r*_0_ corresponds to bond lengths in the experimental structure. The exponent *n* can be estimated by fitting to DFT bands at different volumes. The values thus obtained are listed in Table II.

**Figure 6:**
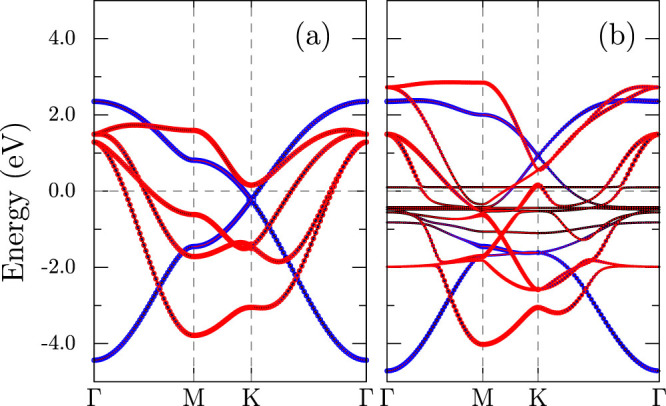
Paramagnetic band structure of MnBi from tight-binding calculations, (a) with Bi-*p* states alone and (b) with both Bi-*p* and Mn-*d* states. The hopping parameters from Table II corresponding to experimental volume *V*_0_ = 97.2Å^3^ and *ζ* = 1.46 are used.

The TB band structure calculated using the derived parameters are shown in Fig. 6 where the important features are found to agree well with DFT bands in Fig. 3. We can also see from Fig. 6(a), where the effect of Mn are ignored, that the large dispersion in Bi-*p* states arise from direct coupling between Bi atoms. The dispersion in 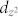 (flat line just above the Fermi level) is not well captured by the model, because we have ignored in-plane Mn coupling.

The magnetism is incorporated in the Hamiltonian via the second term in Eq. 1, which can be written in the Stoner form ignoring any ***k*** dependence, 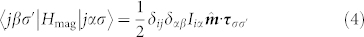
where 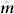 is the direction of magnetization and ***τ*** are the Pauli spin matrices. In this definition, the exchange splitting of each band is equal to the Stoner parameter *I_iα_*. In the present case, there are two parameters *I_d_* and *I_p_*, that correspond to splitting in *d* and *p* states respectively. They can be chosen to fit the experimental magnetic moments and we find, *I_d_* = 4.5 eV and *I_p_* = 0.2 eV, lead to a magnetic moment of 4.1 *μ_B_* per Mn atom.

The final ingredient to the TB Hamiltonian is the spin-orbit coupling term, 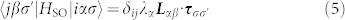
where ***L****_αβ_* are the angular momentum operators. Spin-orbit coupling acts within Mn-*d* and Bi-*p* subspaces and the two parameters are taken to be their atomic values; *λ_d_* = 0.048, *λ_p_* = 1.4 eV respectively.

With the parameters discussed above, the tight-binding model is able to reproduce the correct low temperature magnetic ground state. We get MAE of −2.7 meV, which is overestimated but has the correct sign. In solids, the strength of spin-orbit coupling is weakened by bonding^19^, which may be the source for this error. Thus, we show that a simple model involving only Bi-*p* and Mn-*d* orbitals can be used to study the anisotropy in these systems.

### Lattice dynamics

Experimental measurements on MnBi have found that upon heating, the magnetic moments spontaneously change their direction from *ab* plane to *c* axis. Theoretical studies have suggested that this MAE anomaly can be explained by the lattice expansion upon heating^12^,^13^. The dynamics of the atomic lattice and the magnetic moments can also contribute towards the changes in MAE at high temperatures. To evaluate this, we investigate the lattice dynamics in MnBi in this section.

**Figure 7:**
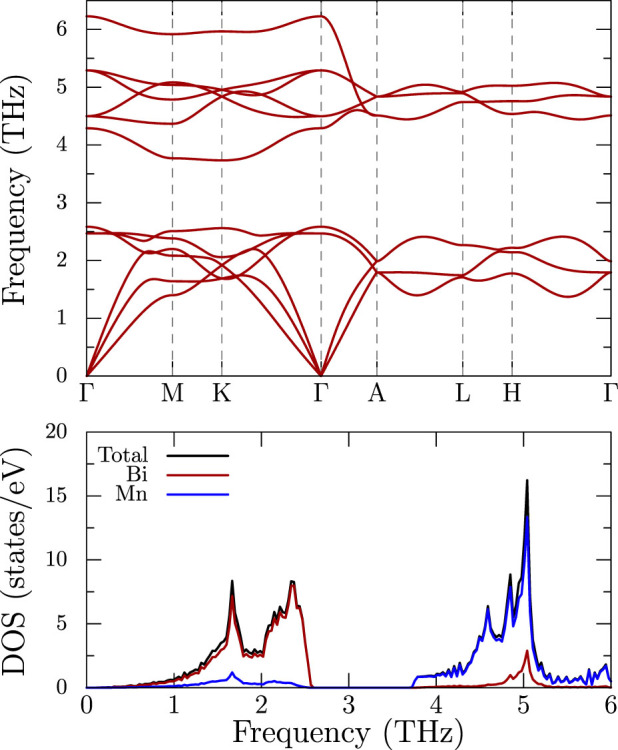
The phonon band structure (top) and the corresponding density of states (bottom) of MnBi in the ferromagnetic structure from first-principles calculations using GGA. The top bands are almost entirely of Mn character, while the low frequency bands are of Bi.

**Figure 8:**
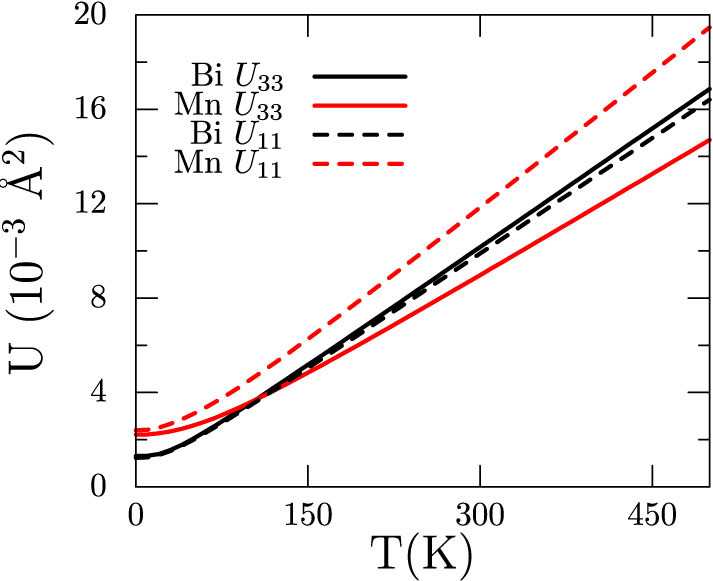
Mean square displacements *U*_11_ (dashed lines) and *U*_33_ (solid lines) for Mn (black) and Bi (red) ions as a function of temperature.

As shown in Fig. 7, the phonon band structure and DOS plots display a near complete separation of the Bi and Mn atom modes, with the Bi modes active between 0 and 2.5 THz and the much lighter Mn modes at higher frequencies between 4 and 6 THz. It is suggestive of the two systems being vibrationally decoupled for nearly all modes except the low frequency acoustic modes, which necessarily involve both atoms. It is of interest to examine this result in light of the mean squared displacements for the two atoms plotted in Figure 8. Despite the general separation of Mn and Bi modes, the mean squared displacements, for both atoms and both directions, are surprisingly consistent, varying by no more than 25 percent at the largest temperatures. This is in agreement with comparable force constants applicable to the Mn and Bi atoms, whose non-acoustic mode frequencies are then split due to the mass difference. Calculated mean square displacements *U*_11_ and *U*_33_ plotted in Fig. 8 show that the displacements of Bi atoms are similar in both *a* and *c* directions. However, the Mn atoms have higher displacements in the *a* direction. At room temperature, the mean amplitude of vibrations is around 0.1 Å.

In general, the high lying Mn optic modes have little dispersion, with the exception of one mode between Γ and A. The low energy modes are of more interest. Surprisingly, in the planar direction Γ to M, the two transverse modes are not nearly degenerate; in fact the higher transverse mode is closer in frequency to the longitudinal mode. Quantitatively, the group velocities along the Γ − *M* are 945, 1800 and 2456 m/s, whereas the velocities along Γ − *A* are 2006, 2006 and 2287 m/s. The difference - nearly a factor of two in the planar transverse velocities, is most unusual, and is suggestive of considerable elastic anisotropy, as these modes sample both planar and axial elastic behavior. No such behavior is present in the Γ to A results, which is as expected for a hexagonal material where the transverse modes are degenerate in this direction.

**Figure 9:**
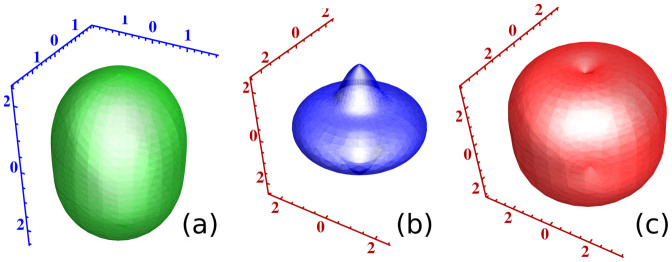
The sound speeds from the first principles calculated elastic constants, in the direction of propagation (in km/sec) of MnBi. Transverse modes (a) and (b), longitudinal mode (c).

Continuing on the theme of elastic anisotropy, in Fig. 9 we present the calculated sound speeds for all three modes, as a function of propagation direction. These sound speeds were generated from first principles calculations of the elastic constants of MnBi using the all electron code WIEN2k, whose calculated elastic constants were found to be (in GPa) *c*_11_ = 69.4, *c*_12_ = 35.7, *c*_13_ = 29.9, *c*_33_ = 55.7, *c*_44_ = 36.9. Several unusual features are found: one transverse mode, (a) has larger sound speeds in the axial direction than in plane, as is evident in Fig. 7. A second transverse mode (b) has a pointed structure along the axial direction, with the remainder of the surface essentially an ellipsoid. Finally, the longitudinal mode (c) appears to show its maximum sound speed in neither the planar nor c-axis direction.

### Effect of lattice vibrations on MAE

At higher temperatures, all the phonon modes are excited and the lattice vibrations can be thought of as isotropic with mean displacements as shown in Fig. 8. Thus, it is possible to get a reasonable estimate of the magnetic anisotropy at different temperatures by averaging over configurations in which the atoms are displaced from their mean positions such that their mean square correspond to Fig. 8 at that temperature.

**Figure 10:**
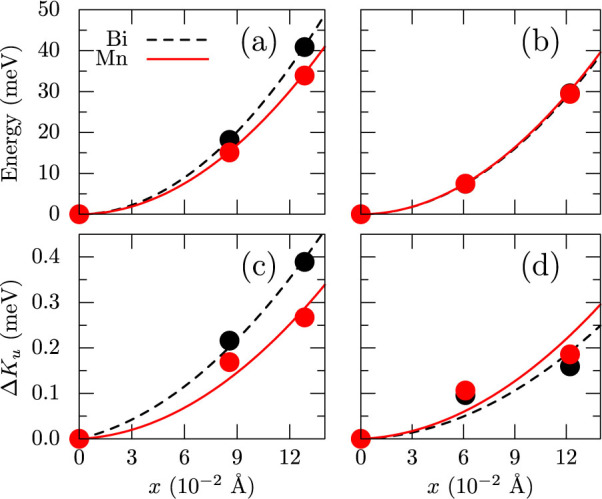
Variation of total energy (a & b) and magnetic anisotropy energy (c & d) as a function of Mn (blue) and Bi (black) displacements from the equilibrium positions (*x*) along in-plane (a & c) and out-of-plane (b & d) directions. The calculations are carried out in a 2 × 2 × 1 experimental structure using GGA. The energy and MAE in the equilibrium structure are taken as the origin.

To make this problem computationally tractable, we consider only a single atomic displacement at a time and only along *a* and *c* directions and calculate the change in anisotropy energy *K_u_* as a function of deviations from the equilibrium positions, denoted as *x*. To minimize the effect of neighbors, we construct a larger 2 × 2 × 1 supercell. Calculated changes in total energies and MAE are shown in Fig. 10, where it can be seen that displacements upto 0.1 Å fall within thermal fluctuations, since *kT* ~ 25 meV. While all deviations increase the anisotropy, in-plane displacements have a stronger effect.

**Figure 11:**
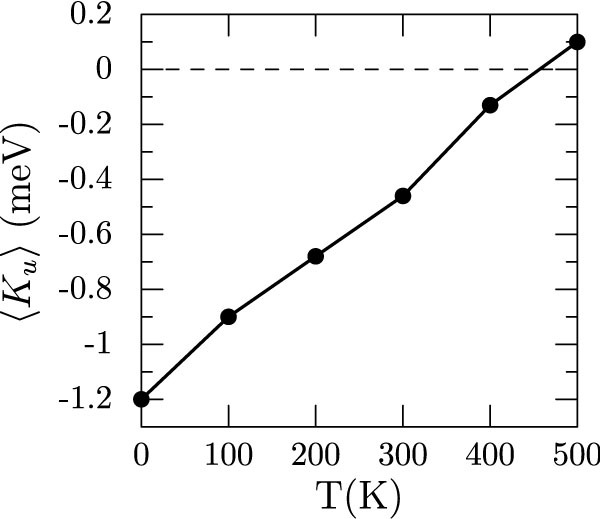
Calculated variation of magnetocrystalline anisotropy *K_u_* per unitcell as a function temperature due to lattice vibrations in the harmonic limit.

From Fig. 10, we see that Δ*K_u_* can be approximated as a quadratic function of *x*, so that *K_u_*(*x*) = *K_u_*(0) + *kx*^2^, where the coefficient *k* depends on the atom types and direction of *x*. Now, to obtain the average MAE, 〈*K_u_*〉, at a particular temperature, we assume the atomic vibrations to be harmonic, i.e., *x*(*t*, *T*) = *a*(*T*) sin *ωt*, where *a*(*T*) is the amplitude and *ω* is the frequency of the vibrations. Then the average is defined as, 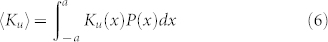
where *P*(*x*) is the normalized probability function of a harmonic oscillator, 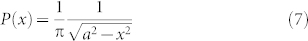
Evaluating the integral yields, 〈*K_u_*〉 = *k*|*a*(*T*)|^2^/2. Taking the |*a*(*T*)|^2^ from Fig. 8 and summing over atoms and directions, we get 〈*K_u_*〉 as a function of temperature which is plotted in Fig. 11. Interestingly, it shows that atomic vibrations alone can result in spin-reorientation, albeit at a much higher temperature of 450 K.

This simplified model shows that the change in MAE upon heating to 300 K, is ~0.8 meV, which is about 64% of the total change observed experimentally. Even though we have ignored all anharmonic effects and considered only isolated displacements, already these calculations suggest that lattice dynamics play an important role in the magnetic behavior of MnBi.

## Discussion

First-principles electronic structure, as well as lattice dynamics calculations have been performed in an attempt to understand the magnetic properties of the ferromagnet MnBi. A tight binding model of this system has also been developed. Our calculations suggest unusual magneto elastic properties in this system. The calculated phonon spectra and sound speeds show anisotropic features, including a transverse mode with nearly double the planar sound speed of the other transverse mode, as well as longitudinal sound speeds which are maximal at non-symmetry locations. All these observations together suggest that MnBi's magnetic interaction with the crystalline lattice is substantially more complex than previously believed. We also find that lattice vibrations contribute significantly to the temperature dependence of magnetic anisotropy and should be taken into account. This rich behavior including both magnetoelastic (bonding related) and magnetostrictive (anisotropy induced) couplings suggests a variety of interesting physical behavior such as temperature dependent magnon-phonon couplings, elastic and phonon anomalies under field and with temperature and potentially opportunities for tuning the magnetic behavior of MnBi. We believe experimental measurements of temperature dependent vibrational spectra are needed to gain further understanding of these issues. We note that, while the results imply that there could be interesting isotope effects on the magnetic behavior, Mn and Bi do not have convenient stable isotopes for such studies.

## Methods

For first-principles calculations, we use DFT method implemented in the Vienna *ab initio* simulation package (VASP)^20^,^21^ with a Γ-centered grid containing more than 11 k points in the Brillouin zone and 400 eV plane-wave energy cutoff. The gradient corrected GGA exchange-correlation functional is used for the calculations, unless otherwise noted^22^. Lattice parameters in Table I, elastic constants and sound velocities are calculated using the all-electron code WIEN2k^23^.

To obtain the phonon band structure shown in Fig. 7, we employed the PHONPY code^24^. It works by doing several total energy calculations on supercells incorporating “frozen phonons,” or atomic displacements dictated by the hexagonal crystal symmetry. Note that spin-orbit coupling is not incorporated in these calculations.

The sound velocities *ν* in the solid are related to the elastic constants *ε* and density *ρ* as, 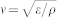. The *ε* is a 6 × 6 matrix with five independent parameters for hexagonal structures. The Christoffel equation is used to solve for the velocities.

In this paper, the magnetic anisotropy is defined as, 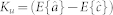 where 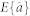 and 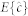 are the total energies of the system with magnetic moments pointing along the directions *a* and *c* respectively.

## Author Contributions

K.V.S. performed calculations of phonons, anisotropy with lattice distortion and constructed the tight binding model. D.P. and D.J.S. performed LAPW calculations. All authors contributed to writing of the manuscript. All authors contributed equally.

## Supplementary Material

Supplementary Information Tight-binding Model

## Figures and Tables

**Table 1:** Lattice parameters *a*, *c* and magnetic moment per Mn and Bi atoms calculated using WIEN2K for three exchange parameters LDA, PBE and PBEsol^25^ in the optimized structure

Exchange	*a* (Å)	*c* (Å)	*m*_Mn_ (*μ_B_*)	*m*_Bi_ (*μ_B_*)
LSDA	4.20	5.54	3.29	−0.08
GGA-PBE	4.35	5.76	3.69	−0.12
GGA-PBEsol	4.28	5.63	3.56	−0.10

**Table 2:** Slater-Koster parameters obtained by fitting the paramagnetic TB bands to that from DFT calculations at different volumes. The distance dependence has the form *t*(*r*) = *t*(*r*_0_)(*r*_0_/*r*)*^n^*

		*t*(*r*_0_)	*t*(*r*_1_)	*t*(*r*_2_)	
	*r*_0_ (Å)	*V*_0_ = 97.5 Å^3^	*V*_1_ = 110 Å^3^	*V*_2_ = 120 Å^3^	*n*
*t_ddσ_*	3.06	0.2	0.15	0.12	4.8
*t_ddπ_*	3.06	0.07	0.04	0.02	6.0
*t_ddδ_*	3.06	0.008	0.005	0.001	6.0
*t_ppσ_*	3.93	1.1	0.9	0.8	4.9
*t_ppπ_*	3.93	−0.25	−0.15	−0.14	11.5
*t_dpσ_*	2.91	0.8	0.7	0.6	3.9
*t_dpπ_*	2.91	−0.2	−0.17	−0.15	4.1

## References

[b1] Guillaud C. Polymorphisme du composé défini MnBi aux températures de disparition et de réapparition de l'aimantation spontanée. J. Phys. Radium 12, 143; 10.1051/jphysrad:01951001202014300 (1951).

[b2] Roberts B. W. Neutron diffraction study of the structures and magnetic properties of Manganese Bismuthide. Phys. Rev. 104, 607; 10.1103/PhysRev.104.607 (1956).

[b3] Yang J. B. *et al.* Magnetic properties of the MnBi intermetallic compound. Appl. Phys. Lett. 79, 1846; 10.1063/1.1405434 (2001).

[b4] Di G. Q., Iwata S., Tsunashima S. & Uchiyama S. Magneto-optical kerr effects of MnBi and MnBiAl films. J. Magn. Magn. Mater. 104–107, 1023; 10.1016/0304-8853(92)90471-Y (1992).

[b5] Chen D. & Gondō Y. Temperature dependence of the magneto-optic effect and resonance phenomena in oriented MnBi films. J. Appl. Phys. 35, 1024; 10.1063/1.1713361 (1964).

[b6] Chen T. Contribution to the equilibrium phase diagram of the Mn-Bi system near MnBi. J. Appl. Phys. 45, 2358; 10.1063/1.1663594 (1974).

[b7] Yang J. B. *et al.* Anisotropic nanocrystalline MnBi with high coercivity at high temperature. Appl. Phys. Lett. 99, 082505; 10.1063/1.3630001 (2011).

[b8] Suzuki K. *et al.* Spin reorientation transition and hard magnetic properties of MnBi intermetallic compound. J. Appl. Phys. 111, 07E303; 10.1063/1.3670505 (2012).

[b9] Harder K.-U., Menzel D., Widmer T. & Schoenes J. Structure, magnetic, and magneto-optical properties of mnbi films grown on quartz and (001)GaAs substrates. J. Appl. Phys. 84, 3625; 10.1063/1.368537 (1998).

[b10] Coehoorn R. & Groot R. A. D. The electronic structure of MnBi. J. Phys. F: Met. Phys. 15, 2135; 10.1088/0305-4608/15/10/009 (1985).

[b11] Sakuma A., Manabe Y. & Kota Y. First principles calculation of magnetocrystalline anisotropy energy of MnBi and MnBi_1−*x*_Sn*_x_*. J. Phys. Soc. Jpn. 82, 073704; 10.7566/JPSJ.82.073704 (2013).

[b12] Zarkevich N. A., Wang L.-L. & Johnson D. D. Anomalous magneto-structural behavior of mnbi explained: A path towards an improved permanent magnet. APL Mat. 2, 032103; 10.1063/1.4867223 (2014).

[b13] Antropov V. P., Antonov V. N., Bekenov L. V., Kutepov A. & Kotliar G. Magnetic anisotropic effects and electronic correlations in MnBi ferromagnet. Phys. Rev. B 90, 054404; 10.1103/PhysRevB.90.054404 (2014).

[b14] Ravindran P. *et al.* Magnetic, optical, and magneto-optical properties of MnX (X = As, Sb, or Bi) from full-potential calculations. Phys. Rev. B 59, 15680; 10.1103/PhysRevB.59.15680 (1999).

[b15] Zhong X.-F., Ching W. Y. & Lai W. Calculation of spin-reorientation temperature in Nd_2_Fe_1_4B. J. Appl. Phys. 70, 6146; 10.1063/1.350024 (1991).

[b16] Dudarev S. L., Savrasov S. Y., Humphreys C. J. & Sutton A. P. Electron-energy-loss spectra and the structural stability of nickel oxide: An LSDA + U study. Phys. Rev. B 57, 1505; 10.1103/PhysRevB.57.1505 (1998).

[b17] Zhiqiang L., Helie L., Wuyan L., Zhi Z. & Qingqi Z. Electronic structure and magnetic properties of MnBi(Al,Nd). Solid State Commun. 79, 791; 10.1016/0038-1098(91)90306-G (1991).

[b18] Brammeier D., Park J.-M., Olson C., Fisher I. & Lynch D. Magneto-optic spectrum and electronic structure of single-crystal MnBi. J. Magn. Magn. Mater. 283, 95; 10.1016/j.jmmm.2004.04.130 (2004).

[b19] Shanavas K. V., Popović Z. S. & Satpathy S. Theoretical model for rashba spin-orbit interaction in *d* electrons. Phys. Rev. B 90, 165108; 10.1103/PhysRevB.90.165108 (2014).

[b20] Kresse G. & Hafner J. Ab initio molecular dynamics for liquid metals. Phys. Rev. B 47, 558; 10.1103/PhysRevB.47.558 (1993). 10.1103/physrevb.47.558 10004490

[b21] Kresse G. Efficient iterative schemes for ab initio total-energy calculations using a plane-wave basis set. Phys. Rev. B 54, 11169; 10.1103/PhysRevB.54.11169 (1996). 10.1103/physrevb.54.11169 9984901

[b22] Perdew J. P., Burke K. & Wang Y. Generalized gradient approximation for the exchange-correlation hole of a many-electron system. Phys. Rev. B 54, 16533; 10.1103/PhysRevB.54.16533 (1996). 10.1103/physrevb.54.16533 9985776

[b23] Blaha P., Schwarz K., Madsen G. K. H., Kvasnicka D. & Luitz J. WIEN2k, An Augmented PlaneWave + Local Orbitals Program for Calculating Crystal Properties (Karlheinz Schwarz, Techn. Universit at Wien, Austria, 2001).

[b24] Togo A., Oba F. & Tanaka I. First-principles calculations of the ferroelastic transition between rutile-type and CaCl_2_-type SiO_2_ at high pressures. Phys. Rev. B 78, 134106; 10.1103/PhysRevB.78.134106 (2008).

[b25] Perdew J. P. *et al.* Restoring the density-gradient expansion for exchange in solids and surfaces. Phys. Rev. Lett. 100, 136406; 10.1103/PhysRevLett.100.136406 (2008). 18517979 10.1103/PhysRevLett.100.136406

