# Supplementary material for: Theoretical study on the role of dynamics on the unusual magnetic properties in MnBi
Source: Sci Rep. 2014 Nov 27;4:7222. doi: 10.1038/srep07222 (PMC4245533; doi:10.1038/srep07222)
Supplement: Supplementary Information — Tight-binding Model [file srep07222-s1.pdf]

# Supplementary material to Theoretical study on the role of dynamics on the unusual magnetic properties in MnBi

K. V. Shanavas, David Parker, David J. Singh

*Oak Ridge National Laboratory, 1 Bethel Valley Road,*

*Oak Ridge, Tennessee 37831, USA*

(Dated: September 14, 2014)

Here, we provide the analytical expressions in terms of the Slater-Koster parameters for the matrix elements of the tight-binding model discussed in Sec. IIC. In our model, we consider only Mn-Mn interaction along $c$. Hence, the matrix part $h_{dd}$ has only $k_z$ dependence and the $5 \times 5$ matrix has the following form,

$$h_{dd} = 2\cos(k_z/2) \begin{pmatrix} t_{dd\sigma} & 0 & 0 & 0 & 0 \\ 0 & t_{dd\delta} & 0 & 0 & 0 \\ 0 & 0 & t_{dd\delta} & 0 & 0 \\ 0 & 0 & 0 & t_{dd\pi} & 0 \\ 0 & 0 & 0 & 0 & t_{dd\pi} \end{pmatrix},$$

in terms of the hopping parameters $t_{dd\sigma}, t_{dd\pi}$ and $t_{dd\delta}$.

Now, each Mn and Bi atoms have six nearest neighbors of the opposite kind, and thus there are four $d-p$ matrices that have similar forms,

$$\begin{aligned} h_{dp}^{13} &= \frac{\exp\left[-i/12(2\sqrt{3}k_y - 3k_z)\right]}{(16+3\zeta)^{3/2}} l_1(g_1, g_2, g_3), \\ h_{dp}^{14} &= \frac{\exp\left[i/12(2\sqrt{3}k_y - 3k_z)\right]}{(16+3\zeta)^{3/2}} l_1(g_1, -g_2, -g_3^*), \\ h_{dp}^{23} &= \frac{\exp\left[-i/12(2\sqrt{3}k_y + 3k_z)\right]}{(16+3\zeta)^{3/2}} l_2(g_1, g_2, g_3), \\ h_{dp}^{24} &= \frac{\exp\left[i/12(2\sqrt{3}k_y + 3k_z)\right]}{(16+3\zeta)^{3/2}} l_2(g_1, -g_2, -g_3^*), \end{aligned}$$

where $\zeta = c/a$ ratio and the functions $l_1$ and $l_2$ are matrices of the type,

$$l_1 = \begin{pmatrix} \sqrt{3}ie_1g_1 & e_1(g_3-g_2) & e_2(g_3+2g_2) \\ -2ie_3g_1 & e_4g_3+e_5g_2 & e_6(g_3-g_2) \\ 4(e_7g_3+e_8g_2) & -2ie_3g_1 & \sqrt{3}ie_6g_1 \\ \zeta(e_7g_3-2e_8g_2) & \sqrt{3}ie_6g_1 & \sqrt{3}ie_9g_1 \\ \sqrt{3}ie_6g_1 & -\zeta(e_{10}g_3+e_3g_2) & e_9(g_3-g_2) \end{pmatrix}$$

$$l_2 = \begin{pmatrix} \sqrt{3}ie_1g_1 & e_1(g_3-g_2) & -e_2(g_3+2g_2) \\ -2ie_3g_1 & e_4g_3+e_5g_2 & e_6(g_2-g_3) \\ 4(e_7g_3+e_8g_2) & -2ie_3g_1 & -\sqrt{3}ie_6g_1 \\ \zeta(2e_8g_2-e_7g_3) & -\sqrt{3}ie_6g_1 & \sqrt{3}ie_9g_1 \\ -\sqrt{3}ie_6g_1 & \zeta(e_{10}g_3+e_3g_2) & e_9(g_3-g_2) \end{pmatrix}$$

The three arguments $g_1, g_2$ and $g_3$ are themselves functions of the crystal momentum components, $k_x$ and $k_y$. They are defined as, $g_1 = \sin(k_x/2)$, $g_2 = \cos(k_x/2)$ and $g_3 = \exp(i\sqrt{3}/2k_y)$. Finally, the parameters, $e_1 - e_{10}$ depend on the Slater-Koster parameters for $d - p$ hopping:

$$
\begin{aligned}
e_1 &= 12\sqrt{3}\zeta^2 t_{dp\pi} + 4(8 - 3\zeta^2)t_{dp\sigma},\\
e_2 &= -48\zeta t_{dp\pi} - \sqrt{3}\zeta(8 - 3\zeta^2)t_{dp\sigma},\\
e_3 &= 2\sqrt{3}(8 + 3\zeta^2)t_{dp\pi} + 4t_{dp\sigma},\\
e_4 &= 12\zeta^2 t_{dp\pi} + 32\sqrt{3}t_{dp\sigma},\\
e_5 &= -12(8 + \zeta^2)t_{dp\pi} + 16\sqrt{3}t_{dp\sigma},\\
e_6 &= -16\sqrt{3}\zeta t_{dp\pi} + 24\zeta t_{dp\sigma},\\
e_7 &= -\sqrt{3}(16 + 3\zeta^2)t_{dp\pi},\\
e_8 &= -\sqrt{3}(8 - 3\zeta^2)t_{dp\pi} + 36t_{dp\sigma},\\
e_9 &= -4(16 - 3\zeta^2)t_{dp\pi} - 12\sqrt{3}\zeta^2 t_{dp\sigma},\\
e_{10} &= -\sqrt{3}(16 - 3\zeta^2)t_{dp\pi} - 48t_{dd\sigma}.
\end{aligned}
$$

For the Bi atom, both nearest neighbor (out-of-plane) and next-nearest neighbor (in-plane) interactions are important. The matrix elements of nearest neighbor term is,

$$
h_{pp} = \frac{2\exp(\frac{-ik_y}{2\sqrt{3}})\cos(\frac{k_z}{2})}{4 + 3\zeta^2}
\begin{pmatrix}
f_2 g_3 + 2f_3 g_2 & 2\sqrt{3}if_1 g_1 & 6\zeta f_1 g_1 \tan(k_z/2)\\
2\sqrt{3}if_1 g_1 & f_5 g_3 + 2f_4 g_2 & 2\sqrt{3}i\zeta f_1(g_2 - g_3)\tan(k_z/2)\\
6\zeta f_1 g_1 \tan(k_z/2) & 2\sqrt{3}i\zeta f_1(g_2 - g_3)\tan(k_z/2) & f_6(g_2 + g_3)
\end{pmatrix}
\quad (1)
$$

Here, the functions $g_1, g_2$ and $g_3$ are as defined above, but the hopping parameters are defined in terms of $p - p$ overlap,

$$
\begin{gathered}
f_1 = t_{pp\pi} - t_{pp\sigma}, \quad f_2 = (4 + 3\zeta^2)t_{pp\pi},\\
f_3 = (1 + 3\zeta^2)t_{pp\pi} + 3t_{pp\sigma}, \quad f_4 = 3(1 + \zeta^2)t_{pp\pi} + t_{pp\sigma}\\
f_5 = 3\zeta^2 t_{pp\pi} + 4t_{pp\sigma}, \quad f_6 = 4t_{pp\pi} + 3\zeta^2 t_{pp\sigma}
\end{gathered}
$$

The next-nearest neighbor Bi-Bi interaction matrix has no $k_z$ dependence and is given

by,

$$h_{pp}^{1} = \begin{pmatrix} h_1 & h_2 & 0 \\ h_2 & h_3 & 0 \\ 0 & 0 & h_4 \end{pmatrix} \tag{2}$$

where,

$$\begin{aligned} h_1 &= 2t'_{pp\sigma}\cos k_x + (3t'_{pp\pi} + t'_{pp\sigma})\cos(k_x/2)\cos(\sqrt{3}y/2), \\ h_2 &= \sqrt{3}(t'_{pp\pi} - t'_{pp\sigma})\sin(k_x/2)\sin(\sqrt{3}k_y/2), \\ h_3 &= 2t'_{pp\pi}\cos k_x + (t'_{pp\pi} + 3t'_{pp\sigma})\cos(k_x/2)\cos(\sqrt{3}k_y/2), \\ h_4 &= 2t'_{pp\pi}\cos k_x + 4t'_{pp\pi}\cos(k_x/2)\cos(\sqrt{3}k_y/2). \end{aligned}$$

The parameters $t'_{pp\sigma}$ and $t'_{pp\pi}$ are the next nearest neighbor Bi-Bi coupling. We can write them as $t'_{pp} = t_{pp}(1/3 + 4\zeta^2)^{n/2}$, with the $n$ values given in Table. II.
